# Herman, Sibley & Co.

**Published:** 1882-01-20

**Authors:** 


HERMAN, SIBLEY & CO., Rochester, New York, and Chicago, Illinois. Seed catalogue—every variety, beautifully illustrated.

